# Transcutaneous Auricular Vagus Nerve Stimulation Triggers Melatonin Secretion and Is Antidepressive in Zucker Diabetic Fatty Rats

**DOI:** 10.1371/journal.pone.0111100

**Published:** 2014-10-27

**Authors:** Shaoyuan Li, Xu Zhai, Peijing Rong, Michael F. McCabe, Jingjun Zhao, Hui Ben, Xing Wang, Shuxing Wang

**Affiliations:** 1 Department of Physiology, Institute of Acupuncture and Moxibustion, China Academy of Chinese Medical Sciences, Beijing, China; 2 Department of Anatomy, Xinxiang Medical University, Xinxiang, Henan Province, China; 3 Department of Anesthesia, Critical Care, and Pain Medicine, Massachusetts General Hospital, Harvard Medical School, Boston, Massachusetts, United States of America; University of Regensburg, Germany

## Abstract

Decreased circulating melatonin is implicated in depression. We recently found that Zucker diabetic fatty rats (ZDF, fa/fa) develop depression-like behaviors and that transcutaneous auricular vagus nerve stimulation (taVNS) is antidepressive in ZDF rats. Here we studied whether the ZDF rats could be used as a depression rodent model and whether the antidepressive effect of taVNS is mediated through modulation of melatonin secretion. Adult male ZDF and Zucker lean (ZL, fa/+) littermates were used. 30 min**-**taVNS procedures (2/15 Hz, 2 mA) were administered once daily under anesthesia for 34 consecutive days in pineal intact ZDF (n = 8) and ZL (n = 6) rats, as well as in pinealectomized ZDF rats (n = 8). Forced swimming test (FST) was used to determine depression-like behavior and ELISA to detect plasma melatonin concentration on day 35. We found that naïve ZDF rats had a longer immobility time in FST and that long-term (34 days) taVNS treatment ameliorated the depression-like behavior. In both pineal intact and pinealectomized ZDF rats, taVNS induced acute melatonin secretion, both during and after the taVNS session. A low melatonin level is related to the poor FST performance in ZDF rats (*R* = −0.544) in contrast to ZL rats (*R* = 0.247). In conclusion, our results show that ZDF rats are ideal candidates of innate depression and that taVNS is antidepressive through triggering melatonin secretion and increasing its production.

## Introduction

A craving for the understanding of major depressive disorder makes the search for additional animal models highly necessary. Proper animal models would accelerate the understanding of the etiology of depression and development of therapeutic approaches. Our recent studies found that Zucker diabetic fatty rats (ZDF, fa/fa) innately develop depression-like behaviors and that transcutaneous auricular vagus nerve stimulation (taVNS) is antidepressive in ZDF rats [1], [2].

Melatonin (N-acetyl-5-methoxytryptamine) is the hormone that functions as the mediator of photoperiodic information to the central nervous system in vertebrates and allows a central circadian regulation of numerous physiological homeostasis including circadian rhythms, sleep, and mood [3]. Importantly, decreased level of circulating melatonin [4], [5] and dysfunction of melatonin receptor type 1 [3] is contributable to depression. On the contrary, complementary with melatonin is associated with reduced depression in rats [4], [5] and patients [6], [7]. However, the melatonin effect was shorter lasting [8], finding approaches that can maintain melatonin secretion will be helpful to clinical patients.

As a kind of complementary and alternative medicine, acupuncture is a generally beneficial, well-tolerated, and safe monotherapy for depression in animal models, clinical patients, and eldercare facility residents [2], [9]. The mechanism for the antidepressive effect of acupuncture may be its stimulation to the vagus nerve [1], [10]–[14]. The taVNS stimulates the afferent auricular branches of vagus nerve that project to solitary nucleus [2]. Neurons in solitary nucleus further project, mono- or multi-synaptically, to the limbic and the autonomic nervous system structures, including the pineal gland, ventral tegmental area, the hypothalamus, amygdala, anterior cingulate cortex, nucleus accumbens, and the lateral prefrontal cortex [2]. Fibers from the solitary nucleus also project to the locus ceruleus and dorsal raphe nucleus, respectively major nuclei related to noradrenergic and serotonergic innervations of the entire brain cortex. The serotonergic, dopaminergic, and noradrenergic systems may be involved in the pathophysiology of depression and in the neuromechanisms of action of antidepressants [2]. However, it is not clear whether the antidepressive mechanism of taVNS is mediated through the modulation of melatonin secretion. Based on the existence of overt depression in Zucker diabetic fatty (ZDF) rats [2] as well as the increasingly prevalent comorbidity of obesity and depression [15], in this study we show that ZDF rats are ideal depression animal model and that the mechanism of the antidepressive effect of taVNS is due to an enhanced tidal release of pineal and extrapineal melatonin.

## Materials and Methods

### Animal model

Male ZDF (n = 29) and ZL littermates (n = 18) were purchased from Vital River Laboratories International Inc. (Beijing, China). The animal room was maintained at 22±1°C artificially lighted from 7:00a.m. to 7:00p.m with light intensity similar to daylight. Littermates from the same or foster mother were housed in one large cage with water and normal food pellets available ad libitum except during taVNS session. The rats entered the experimental procedure at 8 weeks of age and separated into ZDF and ZL groups. At the start of the study the initial body weight was 300±30 grams for ZDF and 200±20 for ZL rats. The rats were raised in groups of three to four. The cages and beddings were changed every other day. The person in charge of the animal's welfare was also the same person who carried out the experiments. We used only male ZDF rats for the study to avoid a possible confounding effect from gender differences on the endogenous melatonin level and other possible hormone variation. The experimental protocol was approved by Institutional Animal Care and Use Committee in China Academy of Chinese Medical Sciences.

### Administration of taVNS

All the time points recorded in this study are in accordance with the taVNS occurrences, i.e. the beginning of taVNS is day 1. Under 2% isoflurane inhalation anesthesia, two opposite magnetic electrodes (+/−) were placed over the right side auricular concha region, two opposite magnetic electrodes (+/−) were placed over the right side auricular concha region, inside and outside respectively so that electronic current can conduct through the tissue, including the vagus nerve fibers. Saline was applied between an electrode and the skin to improve electronic conduction. A 30 min taVNS procedure at a frequency of 2/15 Hz (2 and 15 Hz, switched every second) and an intensity of 2 mA was administered once daily via an electrical stimulator (HANS-100, Nanjing, China). The parameters had been proven effective previously [16]. The procedures were given in the afternoon between 2–5pm for 34 consecutive days. On day 1, 3, and 5, the taVNS was given after last blood sample collection. Auricular margin were used as sham acupoint (ZDF-Sham, n = 4).

### Pinealectomy

To find out the role of pineal gland in depressive behavior and to explore whether there is another melatonin source, the effect of taVNS were also examined in pinealectomized (Px) rats. Pineal gland was removed from rats by referring to a reported method [17]. Briefly, 10 male ZDF rats were weighted and anesthetized intraperitoneally with 50 mg/kg sodium pentobarbital. The animal was fastened to a stereotaxic apparatus; an incision along the skull middle line was made to expose the lambda. The skull at the lambda was opened with a dental drill with a circle bit (0.5 cm Outside Diameter) so that the superior sagittal sinus was directly under view. The superior sagittal sinus was ligated with 5-0 silk suture at the rostral side for two ligations, cut between, and carefully pulled back. The pineal gland, located under the venous sinus, was removed in a single piece using tweezers and the bone fragment was returned to its place, the surgical layers were sutured. Three of the eleven rats exhibiting postoperative poor grooming were euthanatized by intraperitoneal injection of sodium pentobarbital (200 mg/kg) and excluded from further experiments.

### Forced swimming test

FST was performed only once on day 35 between 1 PM and 5 PM. The FST procedure was modified from a previously described method but omitted the pretest session [4], [5], which we found to be a confounding factor to the final results such that the pre-tested rats tended to stay still in formal test and the immobility time was extremely prolonged and obscuring the difference between animal groups. Briefly, a rat was placed in a clear plastic tank (45×35×60 cm) containing 30 cm of water (24±0.5°C) for 5 min. The total duration of non-swimming within the 5-min session was recorded with a stopwatch as immobility scores (in seconds). A rat was judged to be non-swimming when no attempt was made to escape from the tank and the rat was hunched forward (a floating position). Following FST session, a rat was removed from the water tank, dried with paper towel and returned to the home cage. All sessions were observed by the same experimenter blinded to the group assignment to minimize between-experimenter and between-session variations.

### Collection of plasma and ELISA

To determine the acute effect of taVNS on melatonin secretion, plasma samples were collected on days 1, 3, and 5, and at 6 points each day, including immediately before, 15 min during, 0, 30, 60 and 120 min after a taVNS procedure. In order to analyze the chronic trend of melatonin concentration, plasma was collected on day 35, around 22 hours after the last taVNS session.

Under 2% isoflurane anesthesia, 100 µl of blood was collected from tail vein of each rat, each time. The blood sample was centrifuged for 10 min at 110 g, and 60 µl plasma was collected. All plasma samples were stored in −80°C until use. The concentration of melatonin in plasma was analyzed by using enzyme linked immunosorbent assay (ELISA) kit (R&D system Inc. Beijing, China). The plasma melatonin concentration was calculated based on the standard curve and presented in nanograms per liter (ng/L).

**Figure 1:**
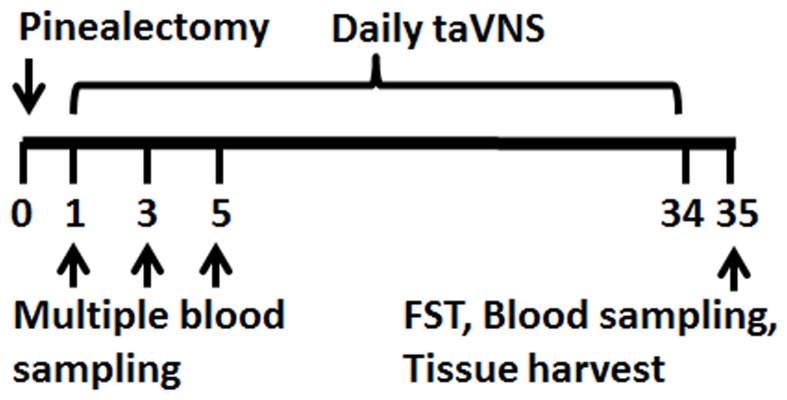
Illustration of experimental time in days. Showing time points for pinealectomy operation, taVNS, blood sampling, FST, and tissue harvest. Pinealectomy operation was done on day 0 (before taVNS sessions). Once daily taVNS administration began on day 1 and continued to day 34. Multiple blood sampling happened on day 1, 3, and 5 of taVNS sessions. On day 35, FST, blood and tissue sampling were done.

Time points for all the above experiments were collectively shown in Figure 1.

### Statistical analysis

By running GraphPad InStat version 3 for Windows, raw data from FST and ELISA were compared using One-way ANOVA to detect differences among treatment groups, followed by Tukey-Kramer Multiple Comparisons Test to determine sources of differences. Data were presented as means ±standard deviation. Differences were considered to be statistically significant at the level of α = 0.05. The correlation between melatonin concentration and immobility time was acquired by linear regression.

## Results

### Tidal melatonin secretion upon taVNS treatment

**Figure 2:**
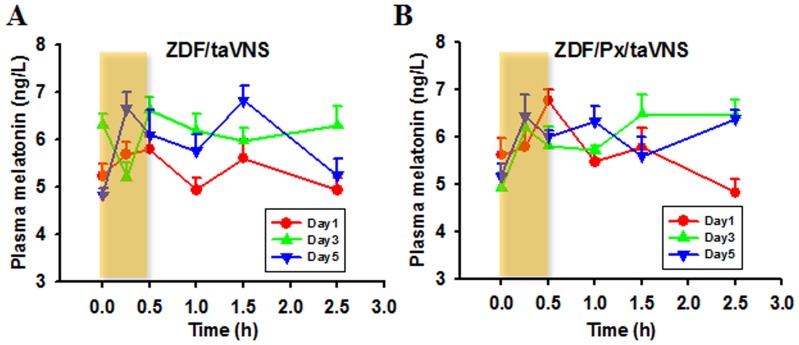
Acute plasma melatonin concentration change upon taVNS. Showing that each taVNS administration (shielded area) would trigger acute and tidal melatonin secretion in pineal intact (A) and pinealectomized ZDF rats (B). Note that the melatonin secretion tides existing both during and after tsVNS session and that the after-taVNS tides tended to be higher (A and B) or more frequent (B) with the increase of treatment days.

The acute effect of taVNS on melatonin level is shown in figure 2. As detected by ELISA on day 1,3, and 5 and from the 6 time points around taVNS procedure, the melatonin concentration changes in a parabolic manner during the taVNS session in ZDF rats. Even after the ending of the taVNS procedure, more parabolic melatonin tides occurs (Fig. 2). As compared between the during- and after-taVNS tides, taVNS session may trigger an acute melatonin increase. The taVNS triggered acute and tidal melatonin secretion exists in pineal intact (Fig. 2A) and pinealectomized ZDF rats (Fig. 2B), indicating that taVNS triggers melatonin secretion from extrapineal sources.

As compared between days, the melatonin tides on day 1 return to pre-stimulation level quickly. The tides tend to be higher or more frequent as each experimental day is completed (Fig. 2A,B).

### Effects of long term taVNS on melatonin secretion and depression-like behavior in ZDF rats

**Figure 3:**
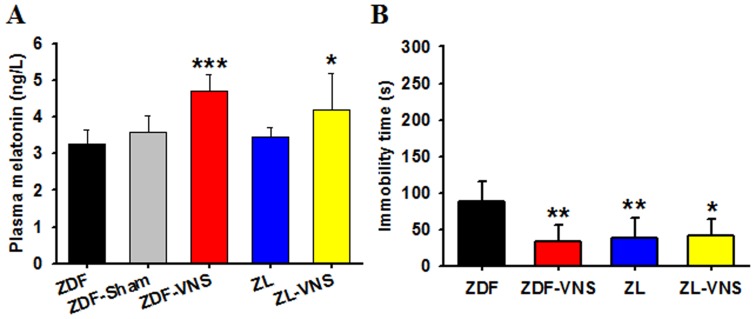
Plasma melatonin concentration and the immobility time in FST on day 35. The total production of melatonin in taVNS treated rats may be higher since the melatonin concentrations were still higher upon sacrifice on day 35, about 22 hours after the last taVNS session on day 34. This may represent the long term effect of taVNS on melatonin secretion (A). In the forced swimming session, a longer immobility time exists in naïve ZDF rats, in contrast to that in taVNS treated ZDF rats or naïve ZL rats (B). *, **, *** *P*<0.05, 0.01, 0.001 *vs.* ZDF, respectively.

As judged upon sacrifice on day 35, about 22 hours after the last taVNS administration, the melatonin concentration in taVNS treated rats is significantly higher (Fig. 3A, Table 1, One-way ANOVA P = 0.0002). Considering the short half-life of melatonin [8], these concentrations represent the long-term effect of taVNS on melatonin level.

**Table 1:** Plasma melatonin concentration in rats on day 35.

Rat groups	Naïve ZDF	ZDF-taVNS	Naïve ZL	ZL-taVNS
Mean	3.273	4.712	3.458	4.2
Standard deviation (SD)	0.385	0.459	0.271	0.999
Sample size (N)	6	8	8	6
Std. error of mean(SEM)	0.157	0.162	0.096	0.408
Lower 95% conf. limit	2.869	4.328	3.231	3.151
Upper 95% conf. limit	3.678	5.095	3.685	5.249
P value vs. Naïve ZDF (Tukey-Kramer)		<0.001	= 0.930	= 0.043
DF	3			
Residual	24			
F	10.050			

As determined by the FST on day 35, the naïve ZDF rats performed poorly in the tank with an immobility time of 87.83±28.03 (mean ± SD, n = 6). This immobility time is much longer than that of ZL rats and of taVNS treated ZDF rats (Fig. 3B, Table 2, One-way ANOVA P = 0.0028).

**Table 2:** Immobility time in rats on day 35.

Rat group	Naïve ZDF	ZDF-taVNS	Naïve ZL	ZL-taVNS
Mean	87.833	33.625	39.25	41.333
Standard deviation (SD)	28.032	22.753	27.639	22.651
Sample size (N)	6	8	8	6
Std. error of mean(SEM)	11.444	8.044	9.772	9.247
Lower 95% conf. limit	58.411	14.600	16.139	17.559
Upper 95% conf. limit	117.26	52.650	62.361	65.108
P value vs. Naïve ZDF, (Tukey-Kramer)		= 0.003	= 0.008	= 0.020
DF	3			
Residual	24			
F	6.231			

The antidepressive effect of taVNS was evaluated in both ZDF and ZL rats. While it is antidepressive in ZDF rats, the taVNS shows no antidepressive effect in ZL rats, possibly due to a relatively shorter immobility time in these rats (Fig. 3, Table 2, P>0.05).

**Figure 4:**
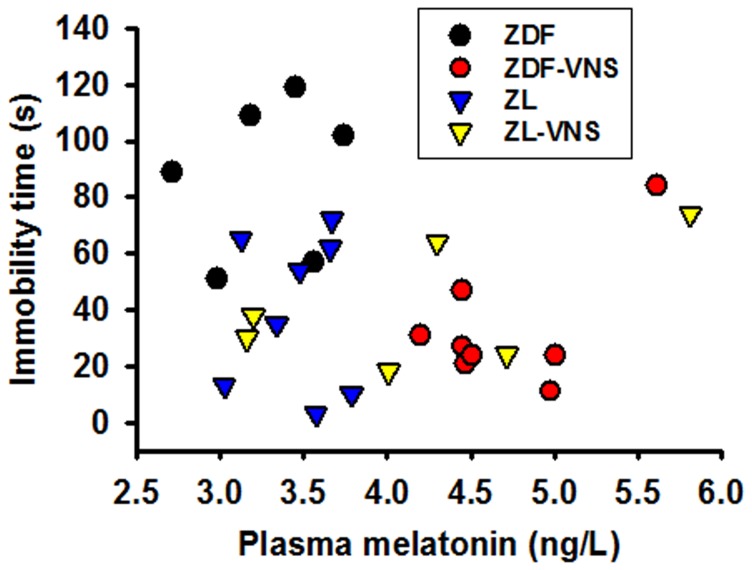
Correlation of melatonin concentration and immobility time in FST on day 35. Linear regression analysis found a strong negative correlation between the plasma melatonin concentration and the immobility time in forced swimming test in ZDF (R = −0.544) but not ZL rats (R = 0.254, also see Table 3).

The correlation between the immobility time and melatonin level on day 35 is shown in Figure 4. The Fig. 4 clearly shows that lower melatonin level is related to the poor FST performance in ZDF rats. As analyzed in linear regression, a strong negative correlation is reached in ZDF (*R* = −0.544), in contrast to ZL rats (*R* =  0.247) in which no overt correlation was shown (Fig. 4, Table 3).

**Table 3:** Correlations between plasma melatonin concentration and immobility time in FST.

Rat group	ZDF	ZL
Expected melatonin level from immobility time	*y* = 4.832−0.0130*x R* = −0.544	*y* = 3.465+0.00752*x R* = 0.245
Expected immobility time from melatonin level	*y* = 152.57−22.766*x R* = −0.544	*y* = 9.481+7.958*x R* = 0.245

## Discussion

ZDF (*fa*/*fa*) rats are widely used for diabetes and obesity research. Here we recommend ZDF rats as good candidates for depression studies based on our results that the ZDF rats have innate depression behavior as detected by FST.

ZDF rats develop type 2 diabetes, in which hyperinsulinemia is usually involved. There are studies suggesting that insulin increases sympathetic nerve activity in obese and diabetic rats by increasing glutamatergic drive to rostral ventrolateral medulla [18], [19]. Moreover, the glutamatergic system has been implicated in the pathophysiology of depression and in the action mechanism of antidepressants [20]. Furthermore, melatonin and dextromethorphan, an N-methyl-D-aspartic acid (NMDA) receptor antagonist, have synergism in another depression rodent model, the Wistar-Kyoto rats [21]. Taken together, these results suggest that a defected interaction between NMDA receptor and melatoninergic system in the brain may play a role in depression. As for the ZDF rats, the depression condition may be worse because they are genetically deficient in leptin receptor and are resistant to leptin, which is an adipocyte-derived hormone with antidepressant-like properties [22].

Under physiological conditions, the main source of melatonin is pineal gland [23]. The pineal gland is innervated by both noradrenergic sympathetic and cholinergic parasympathetic fibers [24]–[26]. While light exposure would activate sympathetic system and reduce pineal melatonin production [25], other environmental factors that modulate the autonomic nervous system or cause epinephrine secretion from adrenal medulla (e.g., distress, insulin-induced hypoglycemia) can override the inhibitory effects of light and accelerate melatonin synthesis [24]. There is also direct evidence that acupuncture elevates melatonin level in pineal gland and subsequently in serum in an animal model of seizures [27].

The taVNS shows antidepressive efficacy even in pinealectomized rats, implying that acupuncture works through the modulation of extrapineal melatonin secretion. Beside the pineal gland, melatonin is also secreted from retina, digestive tract, born marrow, skin, kidneys, ovaries, testis, circulating leukocytes, and many other sources [28]. It should be noted that, these extrapineal melatonin sources not only have a huge total volume, some of them are also rich in melatonin, for example, the melatonin pool of gastrointestinal tract [29] and bone marrow [30]. The concentration of melatonin in both pools surpasses blood melatonin levels by 2 to 3 orders of magnitude and the gastrointestinal tract alone contains over 400× more melatonin than in the pineal gland [29]. A critical factor beneficial for the acupuncture to functionally release melatonin from these extrapineal sources is that they are innervated by vegetative nervous system and the release of melatonin therefrom is out of the photoperiodical regulation [29], [30].

Interestingly, taVNS had differential antidepressive function if compared between ZDF and ZL rats. While taVNS worked well in ZDF rats it had limited efficiency in ZL rats, a similar phenomenon to melatonin treated experimental animals [31]. This may be due to a reduced parasympathetic activity that impedes melatonin secretion in ZDF but not ZL rats.

It should be pointed out that, all the results to date about the vagal nerve stimulation and its triggering and enhancing function to melatonin secretion were reached from preclinical studies in which animals were anesthetized. Thus it is still not clear whether melatonin secretion can be enhanced in a conscious state. Further studies are needed to elucidate the vagal nerve stimulation and melatonin secretion in alert states.

Taken together, the taVNS will trigger a tidal release of melatonin and enhance its production, which in turn will be antidepressive by inhibiting the NMDA induced current and down-regulate the expression of NMDA receptors [4], [32], [33]. Since taVNS induced tidal melatonin release may be maintained for several hour period, it may be used clinically in melatonin deficient patients, such as patients with insomnia and depression.

## Conclusion

Our results support conclusions that ZDF rats are a good depression rodent model and that taVNS is antidepressive by triggering tidal secretion of melatonin and simultaneously increase its production.

## References

[1] Rong P, Fang J, Kong J, Wang L, Meng H, et al (2012) Trancutanous vagus nerve stimulation for the treatment of depression: a study protocol for a double blinded randomized clinical trails. BMC Complement Altern Med12: 255. 10.1186/1472-6882-12-255 PMC3537743 23241431

[2] Liu RP, Fang JL, Rong P, Zhao Y, Meng H, et al (2013) Effects of electroacupuncture at auricular concha region on the depressive status of unpredictable chronic mild stress rat models. Evid Based Complement Alternat Med 2013: 789674. 23431349 10.1155/2013/789674 PMC3570937

[3] Dubocovich ML, Delagrange P, Krause DN, Sugden D, Cardinali DP, et al (2010) International Union of Basic and Clinical Pharmacology. LXXV. Nomenclature, classification, and pharmacology of G protein-coupled melatonin receptors. Pharmacol Rev 62: 343–380. 20605968 10.1124/pr.110.002832 PMC2964901

[4] Wang S, Tian Y, Song L, Lim G, Tan Y, et al (2012) Exacerbated mechanical hyperalgesia in rats with genetically predisposed depressive behavior: Role of melatonin and NMDA receptors. Pain 153: 2448–2457. 23046768 10.1016/j.pain.2012.08.016 PMC3494817

[5] Zeng Q, Wang S, Lim G, Yang L, Mao J, et al (2008) Exacerbated mechanical allodynia in rats with depression-like behavior. Brain Res 1200: 27–38. 18289511 10.1016/j.brainres.2008.01.038 PMC2386964

[6] Maurizi CP (1984) Disorder of the pineal gland associated with depression, peptic ulcers, and sexual dysfunction. South Med J 77: 1516–1518. 6390695 10.1097/00007611-198412000-00010

[7] Cardinali DP, Srinivasan V, Brzezinski A, Brown GM (2012) Melatonin and its analogs in insomnia and depression. J Pineal Res 52: 365–375. 21951153 10.1111/j.1600-079X.2011.00962.x

[8] Nelson E, Panksepp J, Ikemoto S (1994) The effects of melatonin on isolation distress in chickens. Pharmacol Biochem Behav 49: 327–333. 7824545 10.1016/0091-3057(94)90429-4

[9] Lee JH, Choi TY, Lee MS, Lee H, Shin BC, et al (2013) Acupuncture for acute low back pain: a systematic review. Clin J Pain 29: 172–185. 23269281 10.1097/AJP.0b013e31824909f9

[10] Lyons Z, van der Watt G, Shen Z, Janca A (2012) Acupuncture and Chinese herbs as treatments for depression: an Australian pilot study. Complement Ther Clin Pract 18: 216–220. 23059435 10.1016/j.ctcp.2012.06.003

[11] Mischoulon D, Brill CD, Ameral VE, Fava M, Yeung AS (2012) A pilot study of acupuncture monotherapy in patients with major depressive disorder. J Affect Disord 141: 469–473. 22521855 10.1016/j.jad.2012.03.023

[12] Yeung AS, Ameral VE, Chuzi SE, Fava M, Mischoulon D (2011) A pilot study of acupuncture augmentation therapy in antidepressant partial and non-responders with major depressive disorder. J Affect Disord 130: 285–289. 20692042 10.1016/j.jad.2010.07.025

[13] Zhang ZJ, Chen HY, Yip KC, Ng R, Wong VT (2010) The effectiveness and safety of acupuncture therapy in depressive disorders: systematic review and meta-analysis. J Affect Disord 124: 9–21. 19632725 10.1016/j.jad.2009.07.005

[14] La Marca R, Nedeljkovic M, Yuan L, Maercker A, Elhert U (2010) Effects of auricular electrical stimulation on vagal activity in healthy men: evidence from a three-armed randomized trial. Clin Sci (Lond) 118: 537–546. 19895369 10.1042/CS20090264

[15] Preiss K, Brennan L, Clarke D (2013) A systematic review of variables associated with the relationship between obesity and depression. Obes Rev 14: 906–918. 23809142 10.1111/obr.12052

[16] Huang F, Dong J, Kong J, Wang H, Meng H (2014) Effect of transcutaneous auricular vagus nerve stimulation on impaired glucose tolerance: a pilot randomized study. BMC Complement Altern Med 14: 203. 24968966 10.1186/1472-6882-14-203 PMC4227038

[17] Maganhin CC, Simões RS, Fuchs LF, Oliveira-Filho RM, Simões Mde J, et al (2009) Rat pinealectomy: a modified direct visual approach. Acta Cir Bras 24: 321–324. 19705033 10.1590/s0102-86502009000400013

[18] Bardgett ME, McCarthy JJ, Stocker SD (2010) Glutamatergic receptor activation in the rostral ventrolateral medulla mediates the sympathoexcitatory response to hyperinsulinemia. Hypertension 55: 284–290. 20065145 10.1161/HYPERTENSIONAHA.109.146605 PMC2861553

[19] Ward KR, Bardgett JF, Wolfgang L, Stocker SD (2011) Sympathetic response to insulin is mediated by melanocortin 3/4 receptors in the hypothalamic paraventricular nucleus. Hypertension 57: 435–441. 21263116 10.1161/HYPERTENSIONAHA.110.160671 PMC3580160

[20] Mathews DC, Henter ID, Zarate CA (2012) Targeting the glutamatergic system to treat major depressive disorder: rationale and progress to date. Drugs 72: 1313–1333. 22731961 10.2165/11633130-000000000-00000 PMC3439647

[21] Wang S, Zhang L, Lim G, Sung B, Tian Y, et al (2009) A combined effect of dextromethorphan and melatonin on neuropathic pain behavior in rats. Brain Res 1288: 42–49. 19595681 10.1016/j.brainres.2009.06.094 PMC2744035

[22] Guo M, Lu Y, Garza JC, Li Y, Chua SC, et al (2012) Forebrain glutamatergic neurons mediate leptin action on depression-like behaviors and synaptic depression. Transl Psychiatry 2: e83. 22408745 10.1038/tp.2012.9 PMC3298113

[23] Brzezinski A (1997) Melatonin in humans. N Engl J Med 336: 186–195. 8988899 10.1056/NEJM199701163360306

[24] Spence DW, Kayumov L, Chen A, Lowe A, Jain U, et al (2004) Acupuncture increases nocturnal melatonin secretion and reduces insomnia and anxiety: a preliminary report. J Neuropsychiatry Clin Neurosci 16: 19–28. 14990755 10.1176/jnp.16.1.19

[25] Samuels N (2005) Integration of hypnosis with acupuncture: possible benefits and case examples. Am J Clin Hypn 47: 243–248. 15915851 10.1080/00029157.2005.10403638

[26] DeBenedittis G, Cigada M, Bianchi A, Signorini MG, Cerutti S (1994) Autonomic changes during hypnosis: a heart rate variability power spectrum analysis as a marker of sympatho-vagal balance. Int J Clin Exp Hypn 42: 140–152. 8200716 10.1080/00207149408409347

[27] Chao DM, Chen G, Cheng JS (2001) Melatonin might be one possible medium of electroacupuncture anti-seizures. Acupunct Electrother Res 26: 39–48. 11394492 10.3727/036012901816356027

[28] Esposito E, Cuzzocrea S (2010) Antiinflammatory activity of melatonin in central nervous system. Curr Neuropharmacol 8: 228–242. 21358973 10.2174/157015910792246155 PMC3001216

[29] Bubenik GA (2002) Gastrointestinal melatonin: localization, function, and clinical relevance. Dig Dis Sci 47: 2336–2348. 12395907 10.1023/a:1020107915919

[30] Conti A, Conconi S, Hertens E, Skwarlo-Sonta K, Markowska M, et al (2000) Evidence for melatonin synthesis in mouse and human bone marrow cells. J Pineal Res 28: 193–202. 10831154 10.1034/j.1600-079x.2000.280401.x

[31] Paul P, Lahaye C, Delagrange P, Nicolas JP, Canet E, et al (1999) Characterization of 2-[125I]iodomelatonin binding sites in Syrian hamster peripheral organs. J Pharmacol Exp Ther 290: 334–340. 10381796

[32] Kaster MP, Machado DG, Santos AR, Rodrigues AL (2012) Involvement of NMDA receptors in the antidepressant-like action of adenosine. Pharmacol Rep 64: 706–713. 22814023 10.1016/s1734-1140(12)70865-4

[33] Ghasemi M, Raza M, Dehpour AR (2010) NMDA receptor antagonists augment antidepressant-like effects of lithium in the mouse forced swimming test. J Psychopharmacol 24: 585–594. 19351802 10.1177/0269881109104845

